# Bladder Oxidative Stress and HMGB1 Release Contribute to PAR4-Mediated Bladder Pain in Mice

**DOI:** 10.3389/fnsys.2022.882493

**Published:** 2022-05-13

**Authors:** Shaojing Ye, Fei Ma, Dlovan F. D. Mahmood, Katherine L. Meyer-Siegler, Lin Leng, Richard Bucala, Pedro L. Vera

**Affiliations:** ^1^Lexington VA Health Care System, Research and Development, Lexington, KY, United States; ^2^Department of Natural Sciences, St. Petersburg College, St. Petersburg, FL, United States; ^3^Department of Internal Medicine, Yale University, New Haven, CT, United States; ^4^Department of Physiology, University of Kentucky, Lexington, KY, United States

**Keywords:** bladder pain, PAR4, MIF, HMGB1, ROS

## Abstract

Activation of intravesical PAR4 receptors leads to bladder hyperalgesia (BHA) through release of urothelial macrophage migration inhibitory factor (MIF) and urothelial high mobility group box-1 (HMGB1). MIF deficiency and/or MIF antagonism at the bladder block BHA in mice yet the mechanisms are not clear. Since oxidative stress and ERK phosphorylation are involved in MIF signaling we hypothesized that oxidative stress and/or ERK signaling, activated by MIF release, promote intravesical HMGB1 release to induce BHA. We induced BHA by intravesical PAR4 infusion in female C57BL/6 mice. Mechanical sensitivity was evaluated by measuring abdominal von Frey (VF) 50% thresholds before (baseline) and 24 h post-infusion. Intravesical pre-treatment (10 min infusion prior to PAR4) with N-acetylcysteine amide (NACA; reactive-oxygen species scavenger; 3 mg in 50 μl), FR180204 (selective ERK1/2 inhibitor; 200 μg in 50 μl), ethyl pyruvate (EP; HMGB1 release inhibitor; 600 μg in 50 μl), or diluent controls (50 μl) tested the effects of pre-treatment on PAR4-induced BHA. Intravesical fluid was collected after each treatment and HMGB1 concentration was measured using ELISA. Awake micturition parameters (volume and frequency) were assessed at the end of the experiments. Bladders were collected and examined for histological signs of edema and inflammation. Pre-treatment with PBS followed by PAR4 induced BHA in mice but PBS followed by scrambled peptide did not. Pre-treatment with NACA or EP partially blocked PAR4-induced BHA while FR180204 had no effect. A significant correlation between intravesical HMGB1 levels and 50% VF thresholds was observed. All PAR4 treated groups had increased levels of HMGB1 in the intravesical fluid compared to PBS-Scrambled group although not statistically significant. No significant effects were noted on awake micturition volume, micturition frequency or histological evidence of bladder edema or inflammation. Our results show that intravesical antagonism of bladder reactive-oxygen species accumulation was effective in reducing PAR4-induced bladder pain. The correlation between intravesical levels of HMGB1 and bladder pain indicates that released HMGB1 is pivotal to bladder pain. Thus, modulating events in the MIF signaling cascade triggered by PAR4 activation (including bladder oxidative stress and HMGB1 release) warrant further investigation as possible therapeutic strategies.

## 1. Introduction

Interstitial Cystitis/Bladder pain syndrome (IC/BPS) is a clinical complex condition that presents with persistent pain, pressure or discomfort arising from the bladder accompanied by other lower urinary tract symptoms such as frequency or urgency and affects several million women and men in the US (Berry et al., 2011; Suskind et al., 2013). Different classification schemes have been developed yet patients are commonly divided between those having inflammatory disease in the bladder (“classic” IC/BPS with Hunner lesions) and those without (about 90% of patients) (Hanno et al., 2015; Akiyama and Hanno, 2019; Whitmore et al., 2019). Bladder pain/discomfort remains the cardinal symptoms of this disease and currently there is no consistent and effective treatment. Greater understanding of the mechanisms of bladder pain may lead to the discovery of potential new therapeutic targets.

We have studied the mechanisms of bladder pain using a rodent model that results in minimal damage to the organ while still producing bladder pain (Kouzoukas et al., 2015, 2016; Ma et al., 2018, 2019b; Ye et al., 2021). In this model, activation of urothelial protease activated receptors leads to release of urothelial MIF. Released MIF is then responsible for triggering release of urothelial HMGB1 to cause pain (Ma et al., 2017b).

MIF is a pro-inflammatory cytokine that is stored pre-formed in immune and non-immune cells (such as urothelial cells) (David, 1966; Jankauskas et al., 2019). In addition, there is accumulating evidence that MIF is involved in mediating or modulating nociception (Wang et al., 2011; Alexander et al., 2012). There is also ample evidence that MIF is involved in nitro-oxidative stress (Jüttner et al., 1998; Alam et al., 2012; Chuang et al., 2012; Cutrullis et al., 2017). In fact, a recent study showed that MIF induced reactive-oxygen species (ROS) generation, ERK phosphorylation and HMGB1 release *in vitro* (Lv et al., 2016). HMGB1 is nuclear protein recently associated with inflammation and pain (Agalave and Svensson, 2014; Tsujita et al., 2020; Sato et al., 2022).

Interestingly, thrombin induces ROS *in vitro* through PAR4 (Carrim et al., 2015) and activation of PAR1 and PAR4 receptors induced ROS in hepatocellular cancer cells (Mußbach et al., 2015). Therefore based on the evidence just reviewed we decided to investigate whether bladder ROS and ERK phosphorylation also participated in our model of PAR4-induced bladder pain.

The primary objective of these studies was to determine whether intravesical antagonism of ROS, ERK activation, or HMGB1 release had an effect on PAR4-induced bladder pain in mice. Secondarily, we examined the effects of these antagonists on PAR4-induced HMGB1 release in the bladder and the relationship of released bladder HMGB1 and bladder pain.

## 2. Materials and Methods

All animal experiments were approved by the Lexington VA Health Care System Institutional Animal Care and Use Committee (VER-19-005-AF) and performed according to the guidelines of the National Institutes of Health. C57BL/6 female mice (Jackson Laboratory, Bar Harbor, ME) were 12–14 weeks of age at the time of the experiments and were housed in standard rodent cages in rooms with a 14 h on (7 a.m.); 10 h off (9 p.m.) light cycle with *ad libitum* access to food Envigo 2018 Teklad global 18% protein rodent diet and water.

### 2.1. Acute Bladder Hyperalgesia (BHA) Model and Test of Antagonist Pre-treatment

We used intravesical administration of PAR4-activating peptide (PAR4-AP) to elicit acute abdominal mechanical hypersensitivity (interpreted as BHA) as described earlier (Kouzoukas et al., 2015, 2016; Ye et al., 2021). We tested whether intravesical pre-treatment with different antagonists could block PAR4-induced BHA (*N* = 6 mice/group).

Briefly, mice were anesthetized with isoflurane (3% induction; 1.5% maintenance) and transurethrally catheterized (PE10, 11 mm length). Urine was drained by applying gentle pressure to the lower abdomen. Mice received a slow intravesical infusion of PAR4-AP (AYPGKF-NH2) to induce BHA while a corresponding scrambled peptide (PAR4 control; YAPGKF-NH2) served as control. The peptides (100 μM) were dissolved in sterile phosphate-buffered saline diluent (PBS, pH 7.4, 100 μl) and remained in the bladder for 1 h (Kouzoukas et al., 2015, 2016; Ye et al., 2021).

**Figure 1:**
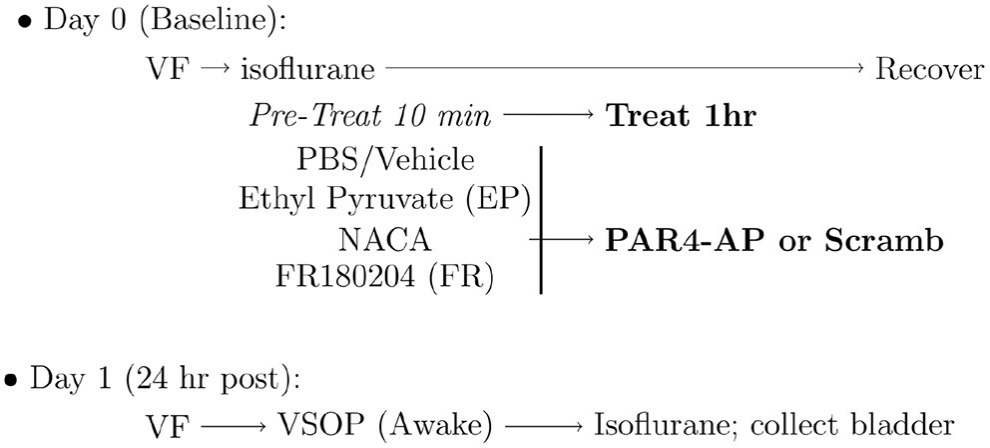
Experimental protocol for induction of bladder hyperalgesia (BHA). Baseline 50% von Frey (VF) threshold was measured before administering intravesical pre-treatments to block protease activated receptor-4 (PAR4)-induced BHA as detailed in the Methods section. NACA, N-acetylcysteine amide; PAR4-AP, PAR4-Activating peptide; VSOP, voided stain on paper.

Pre-treatment to block BHA consisted of slow infusion (50 μl each) of the following substances were administered 10 min prior to activation of PAR4 receptors (Figure 1):

1. N-Acetylcysteine amide (NACA, 3 mg; PBS as vehicle) as a ROS scavenger (Grinberg et al., 2005; Pandya et al., 2014).
2. FR180204 (200 μg; 0.1% methyl cellulose/saline as vehicle) as selective ERK1/2 phosphorylation inhibitor (Ohori et al., 2007).
3. Ethyl pyruvate (EP, 600 μg; PBS as vehicle) to block HMGB1 release (Davé et al., 2009).

The intravesical fluid was collected at the end of the infusion time, treated with phosphatase and protease inhibitors, and stored at −80°C prior to analysis.

### 2.2. Abdominal Mechanical Sensitivity

Mice were acclimated to the testing conditions as follows. Days 1, 2: Acclimation to testing room; mice cage placed in testing room and left undisturbed for 3 h. Days 3, 4: Acclimation to testing chamber; mice placed in testing chamber and left undisturbed for 2 h. Day 5: Acclimation to von Frey monofilaments: mice placed in testing chamber and von Frey monofilaments applied to lower abdominal area. Mice were allowed to rest for 2 days before measuring von Frey baseline threshold as described below.

We tested abdominal mechanical hypersensitivity in mice as previously described (Ma et al., 2018, 2019b; Ye et al., 2021). Briefly, 50% mechanical threshold (Chaplan et al., 1994) was calculated by measuring the response to von Frey fibers (0.008, 0.02, 0.07, 0.16, 0.4, 1.0, 2.0, and 6.0 g) applied to the lower abdominal region. A positive response was defined as any one of three behaviors: (1) licking the abdomen, (2) flinching/jumping, or (3) abdomen withdrawal. Whenever a positive response to a stimulus occurred, the next smaller von Frey filament was applied. Otherwise, the next higher filament was applied. 50% thresholds were measured at baseline (prior to any treatment) and approximately 24 h after bladder pre-treatments and treatments.

### 2.3. Voided Stain on Paper (VSOP): Micturition Parameters in Awake Mice

We measured micturition volume and frequency in awake mice using the VSOP method (Sugino et al., 2008) as described earlier (Ye et al., 2021). Briefly, 24 h after intravesical treatment and after obtaining VF threshold scores, mice were placed in a plastic enclosure individually with freedom to move around and access to water. Filter paper was placed under the cage to collect urine during a 3 h observation period. Micturition volumes were determined by linear regression using a set of known volumes. Micturition frequency is reported as the numbers of micturition per 3 h observation period.

### 2.4. HMGB1 Levels in Intraluminal Fluid

Intraluminal fluid samples were collected at the end of the experiment and immediately centrifuged to remove cells and stored at −80°C until use. The levels of HMGB1 in the intraluminal fluid were assessed using a HMGB1 ELISA kit (MBS2701751, MyBioSource) following the manufacturer's instructions.

### 2.5. Histology

At the end of study mice were anesthetized with 3% isoflurane, bladders were rapidly removed, and segments were placed in 4% buffered formaldehyde for histology. Bladder paraffin sections (5 μm) were processed for routine hematoxylin and eosin (H&E) staining. H&E stained sections were evaluated by two separate experimenters (FM, DM) unaware of the experimental treatment groups and scored separately for edema and inflammation according to the following scale: (0) No edema/no infiltrating cells; (1) Mild submucosal edema/few inflammatory cells; (2) Moderate edema/moderate number of inflammatory cells; (3) Frank edema, vascular congestion/many inflammatory cells, as per our previous studies (Kouzoukas et al., 2015, 2016; Ma et al., 2017a,b, 2019a; Ye et al., 2021).

### 2.6. Reagents

PAR4-AP (AYPGKF-NH2; to elicit BHA) and corresponding scrambled peptide (YAPGKF-NH2; as control) were from Peptides International, Inc. (Louisville, KY). N-Acetylcysteine amide was purchased from Tocris (Minneapolis, MN). FR180204, ethyl pyruvate and methyl cellulose were from Sigma-Aldrich. H&E staining reagents were from Fisher Scientific. Mouse HMGB1 enzyme-linked immunosorbent assay (ELISA, MBS2701751) kits were obtained from MyBioSource, Inc. (San Diego, CA). The rest of the materials used were from Sigma-Aldrich or as described in the methods.

### 2.7. Statistical Analysis

All statistical analyses were performed using R (R Core Team, 2021). Differences in 50% threshold scores were evaluated using a repeated measures 2-way ANOVA with time and treatment as factors (rstatix package). Simple main effects for treatment were assessed at each time point with ANOVA followed by Dunnett's test (using PBS-PAR4 as reference group) if the ANOVA was significant. Changes in micturition parameters, bladder histological scores and intravesical HMGB1 ELISA results were assessed with ANOVA followed by Dunnett's test (using PBS-Scrambled group as reference group) if the ANOVA was significant. A *p* < 0.05 was considered statistically significant. Values reported are mean and ± SEM.

## 3. Results

### 3.1. Effects of Intravesical Treatments on PAR4-Induced BHA

**Figure 2:**
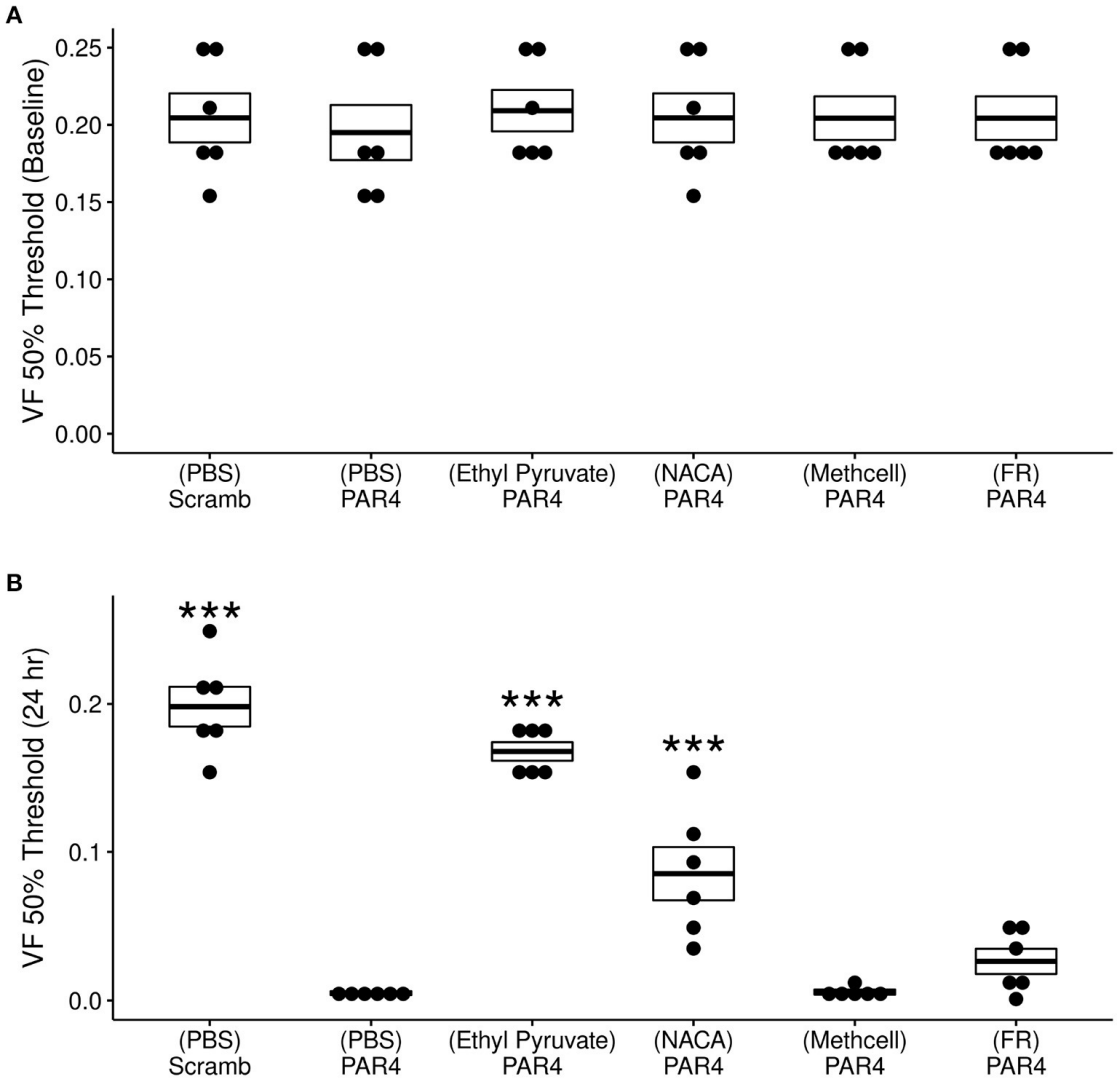
Effects of different treatments (*N* = 6/group) on protease activated receptor-4 (PAR4)-induced bladder hyperalgesia (BHA). **(A)** No difference in 50% von Frey (VF) threshold score from lower abdominal mechanical stimulation using VF monofilaments was observed at baseline across all treatment groups (*F* = 0. 092; ns). **(B)** Significant differences were observed in VF threshold 24 h after treatment *F* = 69.71, *p* = 1.34^−15^. Mice in the (PBS)-PAR4 (pain group) showed a profound decrease in VF threshold score when compared to the (PBS)- Scrambled group (No pain group). Pre-treatment with ethyl pyruvate nearly blocked while pre-treatment with N-acetylcysteine amide (NACA) partially blocked PAR4-induced decrease in VF 50% threshold. Pre-treatment with either methyl cellulose (Methcell) or FR180204 (FR) was not effective in blocking PAR4-induced BHA. ***p < 0.001; ns, not significant at *p* < 0.05.

Our previous studies demonstrated that intravesical PAR4-AP (but not scrambled peptide) induces BHA (Kouzoukas et al., 2015, 2016; Ma et al., 2018, 2019b; Ye et al., 2021). Figure 2 shows our current results on the effects of intravesical treatments on 50% VF threshold at both baseline (Figure 2A) and 24 h post-treatment (Figure 2B). Repeated measures ANOVA showed a significant effect of time (baseline vs. 24 h ; *F* = 14.6, *p* = −2.84^−7^) and treatment (*F* = 602.4, *p* = 2.05^−21^).

No significant differences were observed in 50% VF threshold at baseline across all treatment (Figure 2A). At 24 h, however, significant treatment effects were observed (Figure 2B). Intravesical treatment with PAR4-AP resulted in a profound and significant decrease in the VF threshold at 24 h when compared to the group treated with scrambled peptide (control; Figure 2B, *p* < 0.001). Intravesical pre-treatment with a membrane permeable ROS scavenger (NACA) followed by intravesical PAR4-AP nearly blocked PAR4-induced BHA at 24 h (Figure 2B). Mice pre-treated with ethyl pyruvate (EP; to inhibit HMGB1 release) showed a partial blockade of PAR4-induced BHA at 24 h when compared to the (PBS)-PAR4 group (Figure 2B; *p* < 0.001). Intravesical pre-treatment with methyl cellulose (as vehicle) or with ERK1/2 inhibitor, FR180204 were not effective in preventing PAR4-induced BHA (No significant differences in 50% VF threshold when compared to (PBS)-PAR4 group; Figure 2B). Put together, these results indicate that oxidative stress is an important component of PAR4-induced BHA and suggest the release of HMGB1 may play a role.

### 3.2. Effects of Intravesical Treatments on Released HMGB1

**Figure 3:**
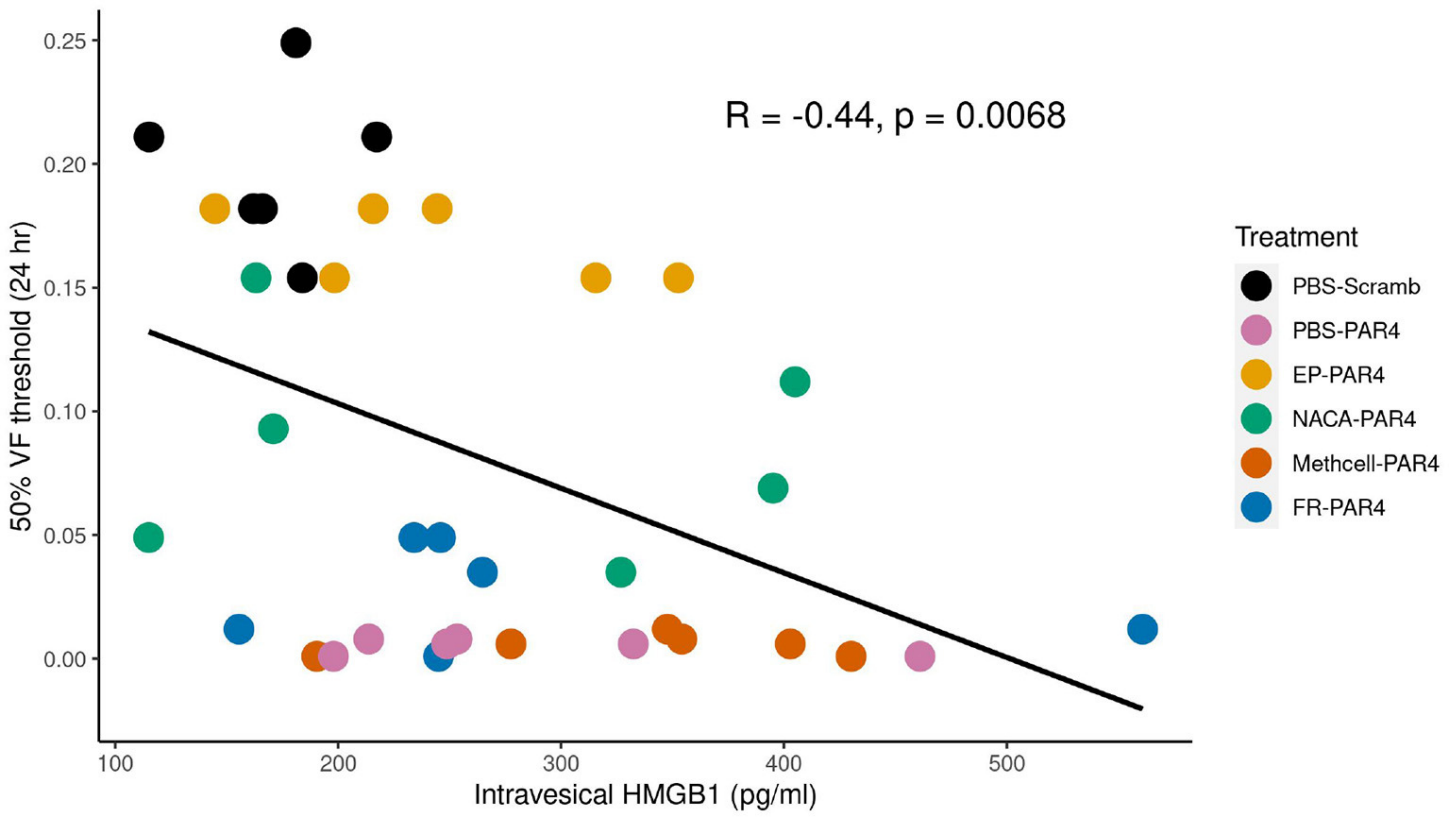
von Frey (VF) threshold is negatively correlated with intravesical high mobility group box 1 (HMGB1) levels in the intravesical fluid. Scatter plot shows a significant negative correlation between intravesical HMGB1 level (x-axis) and 50% VF threshold score (y-axis) indicating that higher HMGB1 concentrations correlate with greater BHA. Treatment groups are color coded as indicated in the legend.

Previous studies using western blot analysis showed that PAR4 activation triggers HMGB1 release from human immortalized urothelial cells (UROtsa) *in vitro* and also *in vivo* (Kouzoukas et al., 2016). Immunofluorescence studies also revealed that urothelial HMGB1 staining is significantly reduced upon PAR4 activation *in vivo*. To further study the relationship between released HMGB1 and bladder pain we measured HMGB1 levels in the intravesical fluid in each of the groups using a specific mouse HMGB1 ELISA kit. All the groups that received intravesical PAR4-AP treatment showed increased levels of HMGB1 in the intravesical fluid when compared to the control group (Scrambled peptide; control; Table 1). These group differences were not statistically significant (*F* = 1.75; ns). However, correlating released HMGB1 levels and 50% VF threshold across all groups showed a significant negative correlation (*R* = −0.44, *p* = 0.006; (Figure 3), suggesting that greater HMGB1 released into bladder cavity leads to greater abdominal mechanical hypersensitivity (more BHA) and further supporting that released HMGB1 from urothelium is an important contributor to PAR4-induced BHA.

**Table 1:** HMGB1 concentration in intravesical fluid after each treatment. Mean ± SEM. N = 6 per group.

**Intravesical pretreat**.	**Intravesical treat**.	**HMGB1 (pg/ml)**
PBS	***Scramb peptide (control)***	171 ± 13.7
PBS	***PAR4-AP***	285 ± 40.1
NACA	***PAR4-AP***	263 ± 52.3
Methcell	***PAR4-AP***	334 ± 35.8
FR	***PAR4-AP***	284 ± 57.5
EP	***PAR4-AP***	245 ± 31.4

### 3.3. Effects of Intravesical Treatments on Micturition Parameters

We evaluated micturition parameters in awake mice with the VSOP method as reported (Ye et al., 2021) in order to exclude the possibility that the treatments may affect bladder function. No statistically significant differences were observed in micturition volume (*F* = 0.43, ns) or frequency (*F* = 1.947, ns) between the control group (PBS pre-treatment and scrambled peptide) and any of the other groups (Table 2). This confirms previous observations that PAR4-induced BHA is not accompanied by micturition changes in this model.

**Table 2:** Effect of intravesical treatments on awake (VSOP) micturition parameters. Mean ± SEM. *N* = 6 per group.

**Intravesical pretreat**.	**Intravesical Treat**.	**Volume (μl)**	**Frequency (voids/3 h)**
PBS	***Scramb peptide (control)***	243 ± 24.0	1.8 ± 0.3
PBS	***PAR4-AP***	233 ± 25.8	2.0 ± 0.3
NACA	***PAR4-AP***	213 ± 61.5	2.7 ± 0.5
Methcell	***PAR4-AP***	278 ± 24.3	1.8 ± 0.2
FR	***PAR4-AP***	258 ± 31.3	1.5 ± 0.2
EP	***PAR4-AP***	226 ± 31.1	2.8 ± 0.6

### 3.4. Effects of Intravesical Treatments on Bladder Inflammation

**Figure 4:**
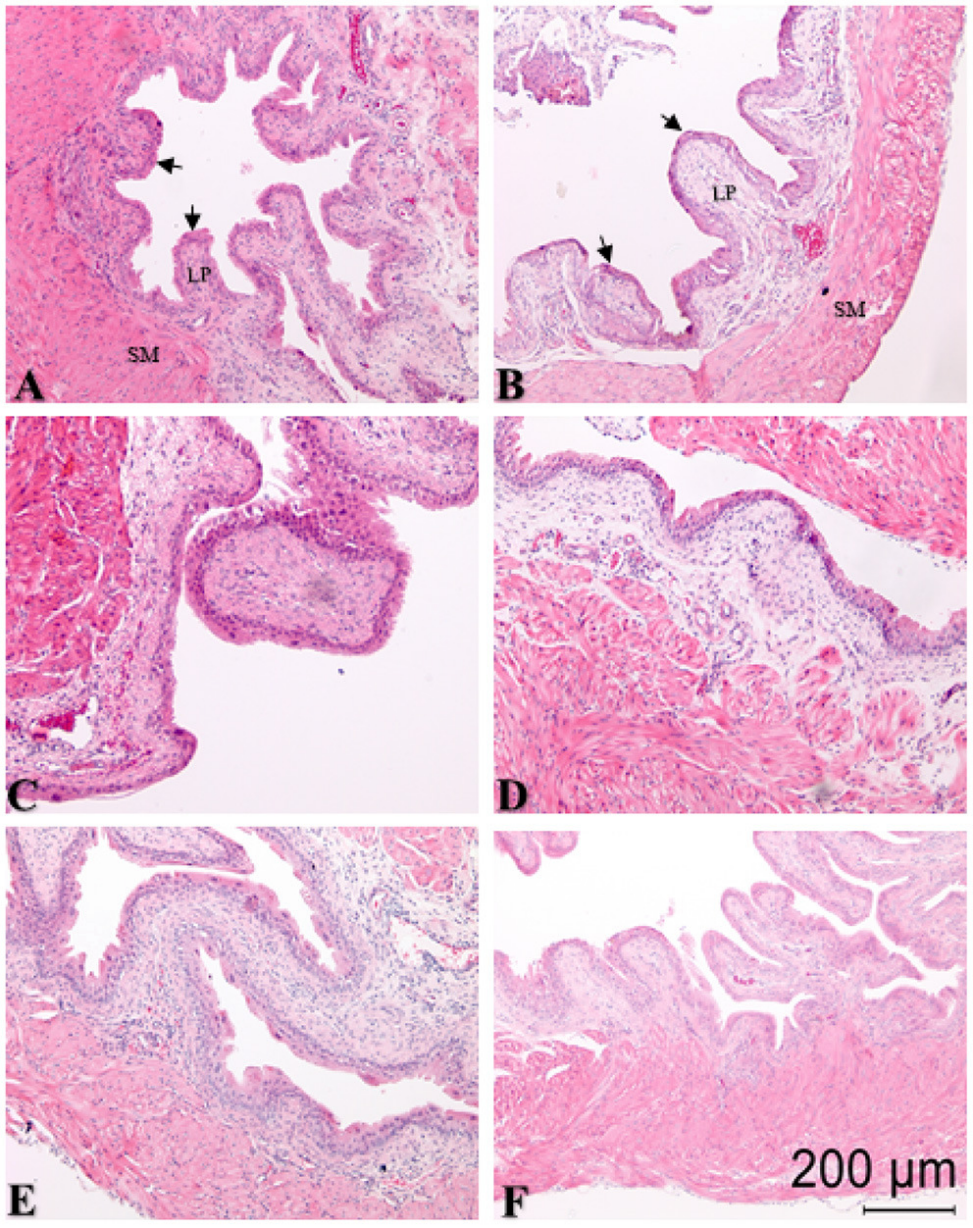
Representative sections of hematoxylin and eosin (H&E) stained bladder cross-sections from all groups (*N* = 6/group) with pre-treatment for 10 min followed by bladder protease activated receptor-4 (PAR4) or scrambled peptide infusion for 1 h. Arrows in panels **(A,B)** show edge of urothelium; lamina propria (LP) and smooth muscle (SM) are also indicated. **(A)** PBS pre-treatment-scrambled treatment; **(B)** PBS pre-treatment-PAR4 treatment; **(C)** N-acetylcysteine amide (NACA) pre-treatment-PAR4 treatment; **(D)** Methylcellulose (Methcell; solvent) pre-treatment-PAR4 treatment; **(E)** FR 180204 (FR) pre-treatment-PAR4 treatment; **(F)** Ethyl pyruvate (EP) pre-treatment-PAR4 treatment. No significant changes in edema (*F* = 1.151, ns) or inflammation (*F* = 0.994; ns) scores were noted in all groups when compared to control (*A*, Table 3; ns, not significant at *p* < 0.05).

We examined the bladder for histological evidence of inflammatory changes at 24 h for all treatment groups. Pre-treatment with intravesical PBS followed by intravesical scrambled peptide (control group) resulted in minimal edema and inflammation (Figure 4 and Table 3). Neither intravesical treatment with PAR4-AP nor any of the other treatments caused a significant increase in edema (*F* = 1.51, ns) or inflammation (*F* = 0.994, ns) in the bladder when compared to the control group (Table 3). This is consistent with previous studies showing that intravesical PAR4 induced BHA only without evidence of inflammatory cell infusion and/or edema in bladder (Kouzoukas et al., 2016; Ma et al., 2017a; Ye et al., 2021) and also suggests that intravesical administration of NACA or EP do not result in urothelial injury or lesion.

**Table 3:** Effect of intravesical treatments on bladder histological changes. Mean ± SEM. *N* = 6 per group.

**Intravesical pretreat**.	**Intravesical treat**.	**Edema**	**Inflammation**
PBS	***Scramb peptide (control)***	0.5 ± 0.3	0.2 ± 0.1
PBS	***PAR4-AP***	1.0 ± 0.3	0.5 ± 0.2
NACA	***PAR4-AP***	0.5 ± 0.2	0.3 ± 0.1
Methcell	***PAR4-AP***	0.8 ± 0.2	0.4 ± 0.1
FR	***PAR4-AP***	0.8 ± 0.3	0.2 ± 0.2
EP	***PAR4-AP***	0.3 ± 0.1	0.2 ± 0.8

## 4. Discussion

PAR4 receptors are expressed in bladder urothelial cells and peripheral nerve endings (D'Andrea et al., 2003). In fact, PAR4 peptides activated bladder afferents in mice causing a significant and sustained increase in afferent firing (Daly, 2011). Thus, intravesical PAR4 peptides may stimulate bladder afferent nerves directly to result in hyperalgesia. However, since PAR4-AP did not cause BHA in MIF KO mice (Ma et al., 2017a) we consider it likely that there is an effect on urothelial cells directly.

Intravesical administration of NACA (an analog of NAC with greater membrane permeability and used as an antioxidant by reducing ROS levels) (Grinberg et al., 2005), partially reduced PAR4-induced BHA (Figure 2B) and supporting our hypothesis. The exact trigger of ROS accumulation remains to be determined since activation of PAR4, MIF and/or TLR4 receptors can induce ROS accumulation (Carrim et al., 2015; Lv et al., 2016; Yin et al., 2019). NAC was reported to inhibit inflammatory bladder pain (Hiramoto et al., 2020) and our current results lend further support for a role for ROS in mediating PAR4-induced bladder pain. Decreased production of ROS has been proposed as a possible novel treatment for pain from IC/BPS (Hagn et al., 2019).

MIF binds to its canonical receptor CD74 to activate signaling pathways through ERK1/2/MAPK phosphorylation (Mitchell et al., 1999) while MIF can also bind other receptors including CXCR4 (Jankauskas et al., 2019). Intravesical blockade of either CD74 or CXCR4 prevented PAR4-induced BHA (Ye et al., 2021). Our current result that pre-treatment with an ERK1/2 specific inhibitor (FR180204) was not effective in blocking PAR4-induced bladder pain (Figure 2B) was not expected. It is possible that solubility issues or current doses of FR180204 were not sufficient to block ERK1/2 phosphorylation. It is also possible that PAR4-induced bladder pain may not be ERK1/2 dependent and may be eliciting ROS production through other signaling pathways.

We previously showed that PAR4 elicits release of HMGB1 from the urothelium *in vivo* and from urothelial cells (primary or immortalized) *in vitro* (Kouzoukas et al., 2016; Ye et al., 2021). EP is well-documented to inhibit active HMGB1 release in different cell systems and injury models (Chung et al., 2008; Davé et al., 2009; Kim et al., 2016; Irie et al., 2017; Seo et al., 2019; Zhang et al., 2019). Intravesical pre-treatment with EP was highly effective in blocking PAR4-induced BHA (Figure 2B) and resulted in a modest reduction in intravesical HMGB1 levels (245 ± 31.4 pg/mL) compared to PBS-PAR4 (285 ± 40.1 pg/mL). This change was not statistically significant (using ANOVA) but may be sufficient to trigger the effect on PAR4-induced bladder pain. Since EP also has anti-oxidant effects (Karabeyoǧlu et al., 2008; Shin et al., 2012) we cannot conclude that effects seen here on PAR-induced BHA are solely due to its ability to block HMGB1 release from the urothelium. However, the correlation between intraluminal HMGB1 levels and BHA (Figure 3) strongly supports a pivotal role for HMGB1 levels in the intravesical fluid (presumably released from the urothelium) in our model of bladder pain (Ma et al., 2017a,b). Other investigators have also noted that HMGB1 plays a role in other (inflammatory) models of bladder pain (Tanaka et al., 2014; Hiramoto et al., 2020) and so it is likely that HMGB1 is involved in bladder nociception.

Patients with IC/BPS are broadly divided into two categories: those with Hunner lesions (detected by cystoscopy) and a robust bladder inflammatory state and those without Hunner lesions or any obvious bladder pathology (approximately 90% of IC/BPS patients) (Whitmore et al., 2019; Homma et al., 2020). In our experiments we rely on a rodent model of bladder pain that results in minimal or no injury to the bladder to isolate pain mechanisms and not in resolution of organ injury.

We noted minimal edema and inflammation during histological examination in the PBS pre-treated and scrambled peptide treated group. This is our “no pain” control group and histological findings likely represents catheter induced changes. None of the other treatments (including intravesical PAR4-AP to induce BHA) caused a significant increase in histological changes over the control group. Moreover, we recently reported that intravesical PAR4 activation was not associated with increases in protein levels or mRNA upregulation of inflammatory cytokines (IL-1β; TNF-α) (Ye et al., 2021), an effect often seen in other inflammatory models of bladder pain (Malley and Vizzard, 2002; Smaldone et al., 2008; Sakura et al., 2009). Thus, several avenues of investigation lead us to conclude that 24 h after PAR4 administration, while mice are still experiencing BHA, there are no demonstrable signs of bladder inflammation. In addition, we did not see any changes in micturition frequency or volume in any of the treatment groups (even those that had untreated BHA) in this study or in any previous study (Kouzoukas et al., 2015, 2016; Ma et al., 2018, 2019b; Ye et al., 2021).

There are other urinary symptoms in IC/BPS (e.g., frequency, reduced micturition volume, urgency) aside from pain at urination. In fact, both Hunner and non-Hunner patients show increased frequency when compared to controls (Peters et al., 2011). Our rodent model can be viewed as limited since we do not find accompanying changes in micturition frequency or volume in mice that are experiencing BHA. On the other hand, this can be viewed as a strength since it focuses on pain mechanisms primarily. Other rodent models commonly used to mimic IC/BPS often result in significant bladder injury with associated increase in edema and inflammation (Bon et al., 1998; Bjorling et al., 2007).

We have evidence that activation of urothelial PAR4 receptors elicits the release of urothelial MIF *in vivo* and *in vitro* (Kouzoukas et al., 2016; Ye et al., 2021). We also reported that activation of PAR4 receptors results in BHA that persisted 24 h post insult (Kouzoukas et al., 2015, 2016; Ma et al., 2018, 2019b; Ye et al., 2021). Released MIF then binds to MIF receptors CD74 and CXCR4 to elicit urothelial HMGB1 release (Ye et al., 2021). Released HMGB1 binds to intravesical TLR4 receptors to mediate bladder pain (Ma et al., 2017b). Our current findings that ethyl pyruvate (known to block active HMGB1 release and also an antioxidant) prevented PAR4-induced BHA confirms our previous results. In addition, we now provide evidence that oxidative stress is involved in PAR4-induced BHA. Oxidative stress has been implicated in the pathogenesis of IC/BPS (Logadottir et al., 2013; Ener et al., 2015). Therefore, further dissecting PAR4-induced signaling pathways may lead to discover new therapeutic targets for bladder pain conditions, including IC/BPS.

## Data Availability Statement

The original contributions presented in the study are included in the article/supplementary material, further inquiries can be directed to the corresponding author/s.

## Ethics Statement

The animal study was reviewed and approved by Lexington VA Health Care IACUC Committee.

## Author Contributions

PV, SY, FM, and KM-S designed the experiments. SY, FM, and DM performed experiments and collected experimental data. PV, SY, FM, and DM performed data analysis. All authors contributed to manuscript preparation and review.

## Funding

This study was funded by NIH (DK121695, PV; AR049610, RB). The material was the result of work supported with the resources and facilities at the Lexington (Kentucky) VA Health Care System.

## Conflict of Interest

The authors declare that the research was conducted in the absence of any commercial or financial relationships that could be construed as a potential conflict of interest.

## Publisher's Note

All claims expressed in this article are solely those of the authors and do not necessarily represent those of their affiliated organizations, or those of the publisher, the editors and the reviewers. Any product that may be evaluated in this article, or claim that may be made by its manufacturer, is not guaranteed or endorsed by the publisher.

## Abbreviations

ANOVA: analysis of variance
BHA: bladder hyperalgesia
ELISA: enzyme-linked immunoassay
EP: ethyl pyruvate
ERK: Extracellular signal-regulated kinases
FR: FR180204
H&E: hematoxylin and eosin
HMGB1: high mobility group box-1
IC/BPS: Interstitial Cystitis/Bladder pain syndrome
IL-1β: interleukin-1β
Methcell: Methylcellulose
MIF: macrophage migration inhibitory factor
NACA: N-acetylcysteine amide
PAR: protease activated receptor
PAR4-AP: PAR4-activating peptide
PBS: phosphate buffered saline
ROS: reactive-oxygen species
TLR4: Toll-like receptor 4
TNF: tumor necrosis factor
VF: von Frey
VSOP: voided stain on paper.
